# Pulmonary Congestion Due to Right and Left Heart Output Mismatching: A Case Report and Literature Review

**DOI:** 10.3389/fphys.2021.665483

**Published:** 2021-04-13

**Authors:** Jing Yuan, Yongjun Li, Jie Sun

**Affiliations:** ^1^Department of Anesthesiology, Zhongda Hospital, Southeast University, Nanjing, China; ^2^Department of Anesthesiology, Lianshui County People’s Hospital, Huai’an, China

**Keywords:** pulmonary congestion, cardiopulmonary bypass, left atrial pressure, bronchospasm, anesthesia

## Abstract

We report a new pulmonary circulation model during cardiopulmonary bypass that is able to cause pulmonary congestion but without left heart failure. This kind of congestion is characterized by right and left heart output mismatching. The pathophysiological mechanism, clinical manifestations, diagnosis, differential diagnosis, and treatment of this pulmonary congestion are reviewed and discussed in the following article.

## Case

A 74-year-old female patient who had mitral valve regurgitation (MR) was admitted to our hospital because of progressive dyspnea for 6 years, and her illness worsened during the previous 2 months. Her body weight and height were 56 kg and 152 cm, respectively. She had been diagnosed with MR for 2 years and had received diuretic and inotropic therapy for a couple of months. After a preoperative physical examination, a systolic murmur (grade 4/6) was identified by cardiac auscultation at the heart apex, and the boundary of heart dullness was found to expand to the left. Transthoracic echocardiography (TTE) revealed a posterior mitral valve leaf prolapse with severe MR. In addition, the patient still had mild-to-moderate tricuspid valve regurgitation, and the pressure gradient was estimated to be 67 mmHg according to the Bernoulli equation. The left ventricular diameter was 52 mm at the section of papillary muscles, and the ejection fraction (EF) value was 74.5%. An electrocardiogram (ECG) showed sinus rhythm with a heart rate (HR) of 73 beats per minute (bpm). A chest computed tomography (CT) scan did not reveal any obvious abnormalities except for a sign of heart shadow enlargement. The patient denied any previous disease history and did not take any medication before this illness. Preoperative anesthetic assessments indicated that she was classified as American Society of Anesthesiologists III status (ASA III) and the surgical risk score was 6 (EuroSCORE of 6). The surgeons decided to replace her mitral valve under cardiopulmonary bypass (CPB).

Before anesthesia induction, the invasive blood pressure (IBP) was 138/62 mmHg after left radial artery A-line establishment. The ECG demonstrated sinus rhythm and the HR was 62 bpm. The pulse oxygen saturation (SpO_2_) value was 94% at room air. After induction of anesthesia with 4 mg midazolam, 50 mg propofol, 0.5 mg fentanyl, and 16 mg vecuronium bromide, the patient underwent tracheal intubation with an ID 7.0 mm tube and was then connected to an anesthetic machine for ventilation. At first, the tidal volume was 450 ml and the respiratory rate was 10 bpm. The inspiratory to expiratory ratio was set at 1:2 without an inspiratory pause. The peak airway pressure was 20 cmH_2_O with the presence of a positive end-expiratory pressure (PEEP) of 5 cmH_2_O. End-tidal carbon dioxide (ETCO_2_) was maintained between 35 and 45 mmHg by subsequently adjusting the ventilation frequency. The patient’s hemodynamics, SpO_2_, and ETCO_2_ were stable and were maintained in the normal range during anesthesia induction. After placement of a transesophageal echocardiography (TEE) probe and a Swan-Ganz catheter, the central venous pressure (CVP), pulmonary artery pressure (PAP), and cardiac output were monitored. The baseline PAP was 56/22 mmHg after Swan-Ganz catheter placement. Anesthesia was maintained with sevoflurane, cis-atracurium, intermittent fentanyl, and the bispectral index (BIS) value was maintained at 40–60 during most time of the surgical procedure.

After about 60 min of mitral valve replacement, the heart began to re-beat successfully after releasing the aortic clamp. The heart contractility seemed good with the help of dobutamine administered at a rate of 5 μg/kg/min. The anesthesiologist found that the peak airway pressure increased gradually from 20 to 38 cmH_2_O after resuming ventilation under the previous ventilator settings. The PAP was not high (10 mmHg) because the patient was still dependent on the CPB machine. The anesthesiologist excluded an inadequate anesthesia depth, airway circuit obstruction, anesthetic machine problems, pleural cavity, or mediastinal abnormalities for a short period of time. Fibrotic bronchoscopy did not reveal bronchus intubation or any obvious secretions. Bronchospasm was initially diagnosed and bronchial dilators together with sevoflurane were administered, but without any obvious effect to relieve the airway pressure. Because bronchospasm usually happens in light anesthetic state and what is more, volatile anesthetics can dilate bronchus directly. Even if it was difficult to listen to breath sound because of aseptic towel, we tried to obtain the breath sound in the two lungs. As pulmonary auscultation did not indicate obvious wet or dry rales and ETCO_2_ waveform did not indicate small airway obstruction, the anesthesiologist began to consider cardiogenic factors. At that time, the IBP was 75/65 mmHg and HR was 90 bpm.

Transesophageal echocardiography examination did not reveal any obvious myocardial contraction abnormalities and the CPB machine perfusion rate was 3.2 L/min. The blood reservoir showed significantly decreased venous return during this period but the surgeons did not note any obvious bleeding around the surgical field. The Swan-Ganz catheter indicated that the right heart stroke volume was much more than the sum of left ventricular outflow tract (LVOT) blood flow volume and the left atrial suction rate. Through echo measurements and calculation, we can get the left ventricle output according to the formula VTI × π × (1/2D_LVOT_)^2^ (VTI, velocity-time integral; D_LVOT_, diameter of LVOT). D_LVOT_ was 0.97 cm and VTI was 5.38 cm. Therefore the stroke volume of left ventricle was almost 4 ml, but the right heart stroke volume was 12 ml through Swan-Ganz catcher measurement. The surgeons confirmed that the left atrial suction tube was working well and excluded an increase of left atrial pressure or left ventricular distension. Due to pulmonary artery blood supply and pulmonary venous return mismatching (Table 1), pulmonary congestion was diagnosed as there was no intracardiac shunt at that time. It seemed that there was no cardiac function problems and there were no effective methods to treat this congestion. In addition, the arterial blood gas (ABG) and body temperature was almost in normal ranges. The medical team decided to wean the patient off CPB gradually. Interestingly, the peak airway pressure began to decrease with progressive weaning, and almost recovered to the baseline level finally. The right heart stroke volume was then closer to the stroke volume which was detected by TEE. After the patient was weaned off CPB, D_LVOT_ was 1.57 cm and VTI was 15.1 cm. Stroke volume of the left ventricle became to be 29.2 ml and the right stroke volume was 30 ml at that moment. The PAP was 45/17 mmHg when the patient was completely weaned off CPB. After that, the airway pressure returned to normal and did not increase again during the rest of operation. Subsequently, the patient recovered very well after the surgery.

**TABLE 1:** Hemodynamic and respiratory data.

	After	Before	After
	induction	weaning-off	weaning-off
SV (mL)	39	12	30
HR (bpm)	65	90	110
LVOT (cm)	1.8	0.97	1.57
VTI (cm)	15.2	5.38	15.1
LVOT flow (mL)	38.6	4.0	29.2
LA suction rate (rpm)		6	
P_peak_ (cmH_2_O)	20	38	22
VT (mL)	450	450	450
I:E ratio	1:2	1:2	1:2
VF (bpm)	10	10	10

*HR, heart rate; I:E ratio, inspiratory to expiratory ratio; LA, left atrium; LVOT, left ventricular outflow tract; P_*peak*,_ peak airway pressure; SV, stroke volume; VF, ventilation frequency; VT, tidal volume; VTI, velocity-time integral.*

## Literature Review

The bronchospasm during general anesthesia may encourage anesthesiologists to consider asthma or anaphylaxis (Maslow et al., 2000; Kuo et al., 2010). But asthmatic patients usually have a previous history of asthma and a medical history of bronchial dilator usage. Anaphylaxis is often accompanied by rush, tachycardia, and hypotension as well as bronchospasm. We treated this patient with extensive bronchodilator regimen but it did not show any obvious effect to relieve high airway pressure.

Consequently, we began to consider another type of pulmonary congestion because we found mismatching of the right and left heart stroke volume (Table 1). Through echo measurements and calculation, we can get the left ventricle output according to the formula VTI × π × (1/2D_LVOT_)^2^ (Tan et al., 2017; Aligholizadeh et al., 2020). D_LVOT_ was 0.97 cm and VTI was 5.38 cm. Therefore stroke volume of the left ventricle was about 4 ml. But the right heart stroke volume was 12 ml through Swan-Ganz catcher measurement. After the patient was weaned off CPB, D_LVOT_ was 1.57 cm and VTI was 15.1 cm. The stroke volume of left ventricle became to be about 29.2 ml, and the right ventricle stroke volume was 30 ml at that moment. Some researchers reported that there was a special refractory bronchospasm during CPB without any obvious reasons or any effective bronchial dilators (Hentz et al., 1984; Kawahito et al., 2001). They were not able to explain it further because traditional bronchospasm seldom happened during deep anesthesia like cardiac surgery anesthesia. The pulmonary congestion we reported and discussed may explain some of the reasons. This type of pulmonary congestion presents a decease of pulmonary compliance, good left heart function without high left atrial pressure or high left ventricle end diastolic pressure (LVEDP), moreover, it shows a mismatching between right and left heart stroke volume.

In healthy human heart and lungs, the right heart cardiac output is nearly equal to that of the left heart, except for a tiny anatomic shunt from the right to the left, which will cause a slight difference between right heart and left heart cardiac output (Ming et al., 2014; Petersson and Glenny, 2014).

**FIGURE 1:**
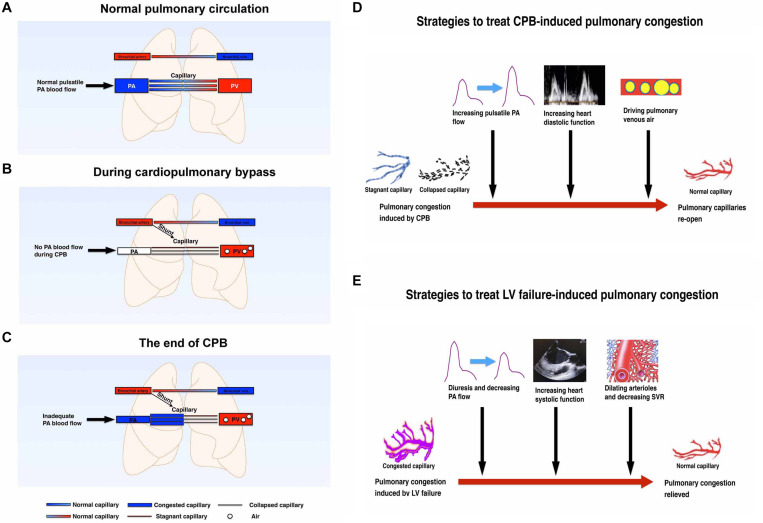
**(A)** Schematic diagram of pulmonary circulation in the normal physiological state without cardiopulmonary bypass. Both lungs are perfused by the pulmonary artery and bronchial arteries. The blood will be oxygenated in the pulmonary capillaries and returned to the left atrium thought pulmonary vein. **(B)** Schematic diagram of pulmonary circulation at the beginning of cardiopulmonary bypass. During cardiopulmonary bypass, the heart stops beating after clamping the aorta. Both lungs are mainly perfused with non-pulsatile bronchial arteries. No blood flows through the pulmonary artery. Some of the pulmonary capillaries will be stagnant or collapsed without normal pulsatile pulmonary blood flow. **(C)** Schematic diagram of pulmonary circulation at the end of cardiopulmonary bypass. After releasing the aortic clamp, some of the pulmonary vessels will be congested because of the following factors: (1) an increase of pulmonary artery flow; (2) capillary flow stagnant and capillary collapse; (3) post-capillary venous air; (4) left atrial diastolic dysfunction. **(D)** Schematic diagram of the strategies to treat CPB-induced pulmonary congestion. CPB can promote pulmonary capillary congestion which may be induced mainly by collapsed and stagnant pulmonary capillaries. Increasing PA pulsatile blood flow, increasing heart diastolic function, and driving pulmonary venous air can treat this congestion effectively and therefore re-open the pulmonary circulation. **(E)** Schematic diagram of the strategies to treat LV failure-induced pulmonary congestion. LV failure can suppress pulmonary venous return and promote pulmonary congestion. Diuresis and decreasing PA flow, increasing heart systolic function, and decreasing SVR can effectively treat this congestion. CPB, cardiopulmonary bypass; LV, left ventricle; PA, pulmonary artery; PV, pulmonary vein; SVR, systemic vascular resistance.

In some congenital heart disease patients, such as atrial septal defect (ASD) or ventricular septal defect (VSD) patients, there will be a mismatching between the right and left heart cardiac output (Yen, 2015). However, there will be a balance between the right heart inflow and outflow tract. In this patient, we found a significant mismatching between the right and left cardiac output. Theoretically, when the intrapulmonary anatomic shunt is neglected, the right heart cardiac output is equal to the left heart output plus left atrial suction rate during CPB. But through calculation, we found that the right heart output was much more than the left heart output. And importantly, this mismatching revealed that the right heart output flow was more than the pulmonary venous returning, which caused pulmonary congestion instead of blood flow mismatching itself. The reasons for this may be explained as follows: (1) during normal heart work, the pulmonary blood is supplied mainly by the pulmonary artery and bronchial artery, and blood will return to the left atrium through the pulmonary vein (Figure 1A). (2) The pressure gradient of the pulsatile pulmonary artery and the related lower pulmonary vein is the main driving force of pulmonary blood flow. However, there are some subtle and reversible changes at the beginning of CPB, and prior to separation from CPB (Figures 1B, and C). It is explained in detail in the following paragraph.

The following changes may have a potential and temporary effect on the pulmonary compliance and airway resistance: (1) the heart stops beating and the pulsatile PAP is missing during CPB, hence the main pulmonary blood driving force will be generated form non-pulsatile bronchial arteries; (2) there will be air filling the pulmonary vein or venules after the left heart opens and then this air will hold part of the pulmonary blood drainage to the left atrium or the CPB machine (Tingleff et al., 1995); (3) pulmonary capillary vessel resistance will be increased with collapses of lung tissue, hypothermia, and nearly stagnant blood flow (Barrios et al., 1959; Kuhn and Turner, 1959; Dekker et al., 2018). In addition, CPB itself may injure the pulmonary endothelium, which has a relatively long-lasting effect of increasing pulmonary arteriolar contractility and promoting pulmonary hypertension (Friedman et al., 1994; Mommerot et al., 2014; Koning et al., 2016). (4) Another important possible mechanism affecting pulmonary vessel resistance is the closed or nearly closed pulmonary microcirculation. Some of the pulmonary capillary vessels will be collapsed and some completely closed during CPB because of hypothermia, non-pulsatile blood flow, thromboxane A2 release, endothelin-1 secretion, complement and neutrophil activation, nuclear factor-kappa B pathway, etc. (Huang et al., 1993; Komai et al., 1993; Ross and DiMarco, 2010; Yang et al., 2015; Karmouty-Quintana et al., 2018). Some of these impacts will last for a couple of days after the surgery but some may recover shortly after CPB [e.g., pulmonary vascular resistance will be increased by hypothermia and/or non-pulsatile blood flow and will quickly recover by rewarming and/or resuming pulsatile blood flow (Halsøy et al., 2016; Taylor et al., 1979]. However, when pulmonary artery flow starts to recover after releasing the aortic clamp, it will be much more difficult for the pulmonary artery blood flow to pass through the pulmonary microcirculation unless these closed or nearly closed microcirculations open again. Micro vessel re-opening and microvascular dilation needs higher pressure and better pulsatile flow besides rewarming. Pulsatile blood flow may have more shear force and will generate more endothelial relaxing mediators to open microcirculation (Nakano et al., 2001; Thacher et al., 2010). As a result, adequately preloaded right heart work is very important not only to re-open these pulmonary microcirculations but also to drive the residual air out of the pulmonary vessels. (5) The last possible reason affecting pulmonary venous drainage is inadequate left atrial diastolic work (Klein et al., 1992; Rossvoll and Hatle, 1993), especially when the left heart cannot fully work very well before complete separation from CPB.

**FIGURE 2:**
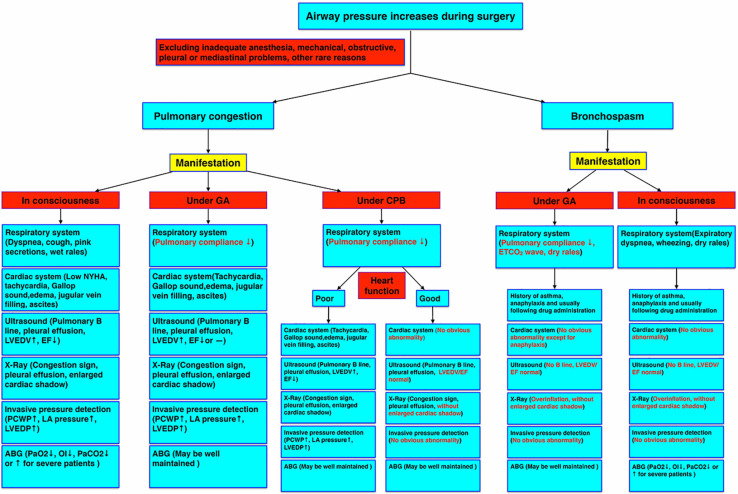
Pulmonary congestion diagnostic criteria under different circumstances. The manifestations of pulmonary congestion under consciousness, general anesthesia, and CPB, and bronchospasm under general anesthesia and consciousness were compared by symptoms and examinations. ABG, arterial blood gas; CPB, cardiopulmonary bypass; EF, ejection fraction; GA, general anesthesia; LA, left atrium; LVEDP, left ventricular end-diastolic pressure; LVEDV, left ventricular end-diastolic volume; NYHA, New York heart association; OI, oxygenation index; PCWP, pulmonary capillary wedge pressure.

We should differentiate this type of pulmonary congestion from bronchospasm, heart failure, allergic reactions, airway secretions, pleural or mediastinal problems, post-CPB lung injury, anesthetic machine malfunction, and protamine adverse effects, etc. (Figure 2). It is reversible only if we treat it correctly. Some researchers have diagnosed it as refractory bronchospasm without any known cause. However, it is not easy to get the direct evidence of bronchospasm by auscultation and difficult to get the typical bronchospasm ETCO_2_ waveform, especially during CPB. Also, this type of bronchospasm is unresponsive to regular bronchodilators, corticosteroids, or inotropic drugs. Left atrial pressure measurement or TEE examination may exclude left heart volume overload or contractile dysfunction. Pulmonary ultrasound and echocardiography are very useful, non-invasive, and effective to diagnose pulmonary congestion and cardiac dysfunction in these patients (Picano and Pellikka, 2016; Miglioranza et al., 2017; Platz et al., 2017, 2019). For example, increased numbers of pulmonary ultrasound B lines are typical signs of pulmonary congestion and some patients may have pleural effusion (Gargani et al., 2015; Facchini et al., 2016). Echocardiography may show increased left ventricular end-diastolic volume (LVEDV), a reduced EF, or preserved EF heart failure (Redfield, 2016; Van Aelst et al., 2018). Another important examination for pulmonary congestion is chest X-ray (Liebman et al., 1978; Costanzo and Fein, 1988), yet chest radiography cannot be obtained in time easily during surgery in most operating rooms. ABG measurement will indicate a decreased oxygenation index (OI) or other abnormalities in conscious patients, whereas it may be well maintained in ventilated patients during anesthesia.

The pulmonary circulation during hypothermic CPB may not be the same as normal circulation during conscious state, so that it is worth while to pay attention to it and calculate specific parameters (Hashimoto et al., 2000; Ege et al., 2003; Buggeskov, 2018). According to the above literature and our experience, resuming of normal heart filling and normal right and left heart work are important to re-open the pulmonary microcirculation, restore pulmonary blood flow, drive pulmonary venous air, and reduce pulmonary congestion (Figure 1D). In addition, we also need to avoid high volume ventilation, hypoxemia, hypercapnia, acidosis, inadequate anesthesia depth, and excessive use of pulmonary vascular constrictors. Because the related factors, which will increase pulmonary vascular resistance, may decrease pulmonary venous returning further (Mahmood and Pinsky, 2018; Pinsky, 2018). But for heart failure-induced pulmonary congestion, the treatment strategy is different (Figueroa and Peters, 2006; Pourtaji et al., 2019; Rivas-Lasarte et al., 2019). Left heart failure, no matter acute or chronic, can suppress pulmonary venous returning and promote pulmonary congestion (Gazewood and Turner, 2017; Rubio-Gracia et al., 2018; Skrzypek et al., 2018; Cops et al., 2019). Diuresis, decreasing pulmonary artery flow, increasing left heart systolic function, and decreasing systemic vascular resistance (SVR) can effectively treat heart failure-induced pulmonary congestion effectively (Figure 1E).

Pulmonary congestion due to right/left heart output mismatching will not happen in every CPB patient and pulmonary tissues are not easily obtained to do histological examination. As a result, there is a barrier to get direct evidence to confirm our hypothesis. However, we have to be cautious about this pathophysiological change to avoid excessive prolonging CPB time when we encounter similar patients. Further studies are needed to confirm our postulate and clarify the specific pathophysiological mechanism in greater detail.

## Author Contributions

JY and YL wrote the manuscript. JS did this anesthesia case, made a thorough analysis of the pathophysiological changes, and finalized the manuscript. All authors contributed to the article and approved the submitted version.

## Conflict of Interest

The authors declare that the research was conducted in the absence of any commercial or financial relationships that could be construed as a potential conflict of interest.
